# Cross-Cultural Adaptation and Preliminary Validation of the Italian Version of the Feeding-Swallowing Impact Survey for both Members of Parental Dyads

**DOI:** 10.1007/s00455-025-10816-4

**Published:** 2025-03-07

**Authors:** Alessandra Baffi, Valeria Crispiatico, Edoardo Nicolò Aiello, Beatrice Curti, Giulia De Luca, Barbara Poletti, Mariagrazia Buratti, Lorenzo Montali

**Affiliations:** 1 AIAS Busto Arsizio Onlus “Annibale Tosi”, Busto Arsizio (VA), Italy; 2 University of Milano-Bicocca, Milan, Italy; 3 Department of Neurology and Laboratory of Neuroscience, IRCCS Istituto Auxologico Italiano, Milano, Italy; 4 Department of Oncology and Hemato-Oncology, Università Degli Studi di Milano, Milano, Italy; 5 Crescere Comunicando, Saronno (Va), Italy

**Keywords:** Caregiver experience, Validation, Adaptation, Feeding disorder, Swallowing, Pediatric

## Abstract

The Feeding/Swallowing Impact Survey (FS-IS) is the first validated instrument to measure the impact of Pediatric Feeding Disorder (PFD) on their caregivers. This study aimed to translate and adapt the FS-IS into Italian (FS-IS-IT) and analyze its reliability and validity, for both fathers and mothers. The FS-IS-IT was developed using Beaton et al.‘s 5-stage process. This cross-sectional study involved 32 dyads of parents of children with PFD and 15 dyads of caregivers of children with developmental disorders without PFD. Twenty caregivers completed the FS-IS-IT questionnaire twice to ensure test-retest reliability. All caregivers completed the Zarit Burden Inventory (ZBI) and the IDDSI Diet Functional Scale for construct validity analysis. ROC analysis was used to evaluating the diagnostic properties of FS-IS-IT in screening between dyads of children with PFD and dyads without these symptoms. The FS-IS-IT was reliable for both fathers and mothers, with satisfactory internal consistency (mothers’ McDonald’s ω=0.93; fathers’ McDonald’s ω=0.94) and test-retest reliability (intraclass correlation coefficient > 0.97). Moderate-to-strong statistically significant correlations (mothers: r(32)=0.73; *p* =.018; fathers: r(32)=-0.42; *p*=.018). r(32)=-0.41; *p*=.018). The FSIS-IT was featured by optimal diagnostics (mothers: AUC=0.97; fathers: AUC=0.94), a cut-off of 1.58 for mothers and 1.65 for fathers has shown good specificity and sensitivity. The FS-IS-IT is a reliable and valid tool for the assessment of the impact of PFD and shows optimal diagnostic properties.

## Introduction

A Pediatric Feeding Disorder (PFD) is defined as “impaired oral intake that is not age-appropriate and is associated with medical, nutritional, feeding skill, and/or psychosocial dysfunction” [1]. This definition includes children with both ‘feeding disorders’ and ‘swallowing disorders’ (i.e. dysphagia). Children with feeding/ swallowing disorders are at risk of malnutrition, poor hydration, respiratory problems, and reduced quality of life [2].

PFDs have varying presentations, severity, and frequency, and different treatment strategies are available [3]. According to Linscheid et al. [4], feeding disorders are estimated to affect between 25% and 45% of typically developing children and up to 80% of children with developmental disabilities. On the other hand, the incidence of dysphagia remains unknown, although studies suggest that the prevalence of swallowing dysfunction has been increasing [5–6]. It is worth noting that infant and child survival rates have improved in recent years due to medical and technological advances [7], increasing the number of children with complex problems. For example, children born prematurely with very low birth weight or neurological impairment often have problems with swallowing and feeding [8]. Various etiological conditions can cause feeding/swallowing difficulties, including but not limited to neurological disorders (cerebral palsy, mental retardation, developmental delay), congenital (e.g. tracheoesophageal fistula) or acquired (e.g. tracheostomy, paralysis or paresis of the vocal cord), genetic diseases (chromosomal, e.g. Down syndrome, syndromes or congenital metabolic disorders) [9] and developmental autism spectrum disorders [10].

It is important to note that PFD can affect not only the child but also the parents. Providing adequate nutrition to children is a fundamental responsibility of parents and an expression of love. Parents who care for children with complex medical conditions, such as feeding disorders and dysphagia, may face a significant caregiving burden derived from mealtimes and nutrition, which can become increasingly stressful over time [2, 11–12]. These children may require adapted feeding strategies, such as consistent food preparation, specific positioning, and the use of special equipment. These challenges can affect daily life [13–14]. Additionally, the presence of PFD may cause anxiety about their child’s weight gain and the risk of unsafe swallowing, leading to feelings of inadequacy and difficulty in entrusting their child’s feeding to others. Restrictions on dining out and participating in social events, such as weddings and parties, may restrict opportunities for social interaction. Special dietary requirements and needing to be present when eating can also increase the financial burden on the family.

These factors can have an impact on the caregiver’s health, psychological well-being, and relationships, as well as the family as a whole. Therefore, it is crucial to provide support to caregivers to maintain their well-being.

It is widely acknowledged in the literature that caregiving, including child-rearing, has traditionally been associated with women’s roles in all cultures and contexts [15]. This feminization pattern of caregiving is also observed among mothers whose children have a disability or chronic health problem [16–17]. Therefore, a major maternal involvement in the management of feeding difficulties has been observed [18]. For this reason, studies of caregiving have traditionally focused more on women’s experiences [19–20], just as validated assessment tools tend to involve mainly or exclusively the mother figure. Also, mothers are primarily involved in counselling and rehabilitation treatment. However, recent evidence suggests that fathers are increasingly involved in caring for children with disabilities, sharing the responsibility with mothers to varying degrees [15, 21]. Therefore, it is important to investigate whether feeding and swallowing difficulties have different impacts on parent dyads.

The FS-IS is the first validated instrument to measure the impact of children’s feeding/swallowing problems on caregivers. It comprises 18 items divided into three subscales: (1) The Daily Activity subscale assesses the impact of managing swallowing and feeding difficulties on a daily basis (5 items); (2) The Worries subscale evaluates the caregivers’ concerns for the child’s health and well-being (7 items); (3) The Feeding Difficulties subscale assesses the challenges associated with managing eating or swallowing difficulties (8 items). Each statement is scored using a five-stage Likert scale, ranging from 1 = “never” to 5 = “almost always”. The score for each subscale is calculated by adding the Likert scores for the items in that subscale and dividing by the number of items answered in that subscale. The original English version [22] has been translated into multiple languages, including Turkish [23], Persian [24] and Brazilian Portuguese [25]. Notwithstanding the fact that the original English version [22] was developed for infants (mean age: 14 months) with heterogeneous pathologies, subsequent cross-cultural validated versions included samples of older children [23, 25] or children with specific pathologies, such as cerebral palsy [23]. Also, it has been shown to be sensitive in detecting changes in caregiver responses to cleft lip and palate interventions [26].

Given the lack of comparable dysphagia-specific tools to assess caregiver burden in children with PFD in Italy, the aims of the present study were (1) to translate and cross-culturally adapt the FS-IS into Italian (FS-IS-IT), (2) to assess the psychometric properties of the FS-IS-IT in terms of reliability (internal consistency, test-retest reliability), validity (criterion and convergent validity) and diagnostic properties (known group validity) for both members of the dyad (mothers and fathers). The validation of the Italian version of the FS-IS is an important step towards improving clinical practice and research. It will allow the impact of the disorder on caregivers to be assessed and the effectiveness of targeted counselling and psychological treatments to be evaluated. In addition, the FS-IS-IT has the potential to support cross-cultural comparisons of clinical and research findings related to feeding and swallowing difficulties experienced by parents in Italian-speaking countries and communities elsewhere.

We hypothesized that PFD would have a differential impact on parents, resulting in higher levels of distress for mothers. Based on previous validation studies [23–26], we expected that the FS-IS-IT would demonstrate satisfactory psychometric properties for both subgroups.

## Materials and Methods

### Study Design

This prospective, non-randomized cross-sectional study with controls was conducted in accordance with the Declaration of Helsinki and was previously approved by Ethics Committee of University of Milano-Bicocca (protocol number: RM-2023-716). The authors of the original English version of the FS-IS granted consent for this study to be conducted.

The study was completed using the COSMIN checklist as a reference for selecting health measurement instruments [27].

### Participants

Both parents of children with feeding/swallowing disorders were enrolled by convenience sampling at AIAS Busto Arsizio Onlus between January 2023 and January 2024. The inclusion criteria were: (1) both parental dyads (i.e. mother and father) must accept enrollment in the study; (2) the child must have a diagnosis of a neurological condition (cerebral palsy, mental retardation, developmental delay), or congenital condition (e.g. tracheoesophageal fistula) or acquired (e.g. tracheostomy, paralysis or paresis of the vocal fold), genetic/chromosomal anomaly ( e.g. Down syndrome, syndromes or congenital metabolic disorders) [9]; (3) being a parent of a child aged ≤ 12 years; (4) according to clinical examination by an expert speech and language therapist (SLT) with a postgraduate masters’ degree in deglutology, in addition to training in PFD and a minimum of five years’ experience in the field, the child must have feeding or swallowing problems; (5) participants were required to have adequate ability to read or listen to Italian in order to understand the purpose of the research; (6) parents were required to be over 18 years of age. The study comprised parents who had previously received an intervention or support with regard to feeding or dysphagia, as well as those who were being visited for the first time.

To test discriminant validity, parents of controls, i.e. parents of a child with neurological, anatomical, or congenital disorders but without feeding or swallowing disorders, were enrolled. A convenience sample was recruited at the same Centre. The inclusion criteria for controls were: (1) parents of a child with congenital or acquired neurological, anatomical, or other disorders [9]; (2) children without reported signs or symptoms of feeding or swallowing disorders by parents or medical team. In particular, the children exhibited no signs of dysphagia or feeding disorders at the assessment conducted by an expert SLT. In addition, the parents did not report any issues related to feeding to the medical team. They confirmed that their child did not require any type of diet modification and that they did not exhibit phobic attitudes towards food; (3) children must have a diet without changes in consistency; (4) parents over 18 years of age were required.

## Materials

### Zarit Burden Interview

Parents completed the Italian- validated version of the Zarit Burden Interview (ZBI) [28]. The ZBI is a reliable and validated tool designed to measure the impact of the patient’s disability on the carer’s quality of life, psychological distress, guilt, financial difficulties, shame, and social and family difficulties. The questionnaire is composed of 22 items, ranging from 0 (never) to 4 (almost always), based on the degree of agreement with each item, and can be used as both a self-report and a structured interview. The questionnaire is straightforward to administer and requires the caregiver to respond on a Likert scale. Scores range from 0 to 88, with higher scores indicating a greater level of burden experienced by the caregiver due to the subject’s condition. The items aim to analyze the impact of the patient’s disability on the carer’s quality of life, psychological distress, guilt, financial difficulties, shame, and social and family difficulties.

### IDDSI Functional Diet Scale

**Fig. 1:**
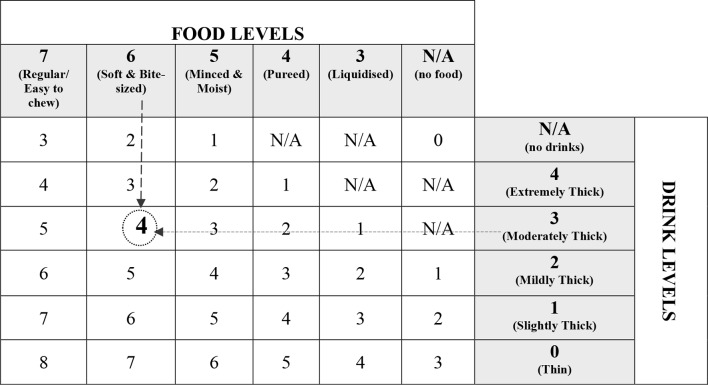
Scoring table for the IDDSI Functional Diet Scale. To determine a patient’s IDDSI-FDS score, the physician must identify the intersecting cell in the column indicating the patient’s food texture recommendation and the row indicating the patient’s beverage consistency recommendation. To illustrate this, consider a patient with a recommendation for level 6 (Soft and small) and level 3 (Moderately thick). In this case, the intersecting cell would indicate a score of 4 on the IDDSI Functional Diet Scale, as illustrated by the dashed line arrows and the square. N/A, not applicable

To evaluate the dietary habits of children, an SLT should complete the IDDSI Functional Diet Scale [29] based on a mealtime observation. This scale was developed using a matrix similar to a mileage chart that was created as an adjunct to the IDDSI framework [30] (see Fig. 1). The matrix captures the degree of dietary texture restriction recommended for a patient based on the assessment of a qualified therapist. The scale ranges from 0 (nothing by mouth) to 8 (no dietary restriction). Therefore, a lower IDDSI Functional Diet Scale score indicates tighter dietary texture restrictions.

### Procedures

The parents provided sociodemographic information and details of the patient’s feeding and swallowing history, including the time of onset of difficulty with food and liquids, as well as any signs of feeding and swallowing disorders. A SLT (first author: AB) followed a script to explain the project to the caregivers and provide them with specific instructions on completing the forms. The researcher was also available to answer any questions.

The caregiver completed the FS-IS-IT and the Italian version of the ZBI [28] using pencil and paper. Additionally, an experienced SLT classified the children’s diet based on the IDDSI Functional Diet Scale [29] based on a mealtime observation. Controls were assigned a IDDSI Functional Diet Scale level of 8.

### FS-IS-IT Development

The FS-IS questionnaire [22] was adapted it to the Italian language and culture, following the five-step process outlined by Beaton et al. [31]: (I) *Forward translation*: an expert bilingual panel of 4 SLTs independently translated the items of the original questionnaire into Italian, prioritizing conceptual over literal translation. The first author, a deglutologist SLT with experience in PFD (AB) and a Ph.D. psychologist (ENA) collected the translated versions and prepared the synthesized version (II) *Synthesis*: a final Italian consensus version was achieved by addressing idiomatic, semantic, and conceptual issues; (III) *Back translation*: the questionnaire was translated back into English by independent professional translators who had no prior knowledge of the FS-IS questionnaire and no experience in dysphagia or feeding disorders. The translators checked all reports for conceptual and cultural equivalence; (IV) *Expert committee review*: This version was then revised by a panel of experts (2 SLT, two psychologists, one cultural-linguistic mediator). Following this, a group of 14 deglutologists evaluated the revised version to assess face and content validity using a 1-4-point ordinal scale. The scale ranged from 1, indicating ‘not relevant’, to 4, indicating ‘very relevant’; (V) *Pretesting*: to verify comprehension of the meaning and wording of test items, the final version was administered to a subgroup of 16 caregivers. Semi-structured cognitive debriefing interviews were conducted with caregivers of children with PFD to determine the clarity, relevance, and applicability of the draft FS-IS-IT. Parents were included according to the same criteria as previously outlined. The parents were asked to complete the questionnaire in the presence of a researcher with a degree in SLT and psychology who made detailed notes about any hesitations or difficulties experienced by respondents. Guided by a semi structured interview schedule, the interviewer asked about the problems observed and specific questions about the suitability of the questionnaire content. Parents were encouraged to provide feedback on the items and instructions, and whether any aspects of their experience were not covered by the questionnaire. It is noteworthy that no significant recommendations were proffered during the pilot testing phase. This iteration was subsequently submitted to the primary author of the FS-IS for analysis and approval to advance with the adaptation process.

## Statistics

### Background Analyses

Linear model assumption checks were performed on raw variables both by assessing skewness and kurtosis values (judged as abnormal if >|1| and >|3|, respectively) [32] and by visually inspecting histograms and Q-Q plots. Accordingly, either parametric or non-parametric statistics were run in order to test the associations/predictions of interest.

To test whether parents’ educational levels could represent a confounder of their FS-IS-IT-based ratings, both the FS-IS-IT Total score and its subscales were correlated with education *via* Pearson’s/Spearman’s coefficients separately for mothers and fathers.

Mothers and fathers were compared on meal assistance rates, as well as on FS-IS-IT and ZBI scores, *via* dependent-sample *t-*tests/Wilcoxon’s tests (for continuous variables) or χ^2^ tests (for categorical variables), separately for children with and without PFD (PFD+; PFD-). Additionally, mothers and fathers of PFD + children were compared to mothers and fathers of PFD- children on the same variables by means of independent-sample *t-*tests/Mann-Whitney’s tests (for continuous variables) or χ^2^ tests (for categorical variables).

The agreement rate between mothers’ and fathers’ scores on the FS-IS-IT was investigated by comparing mothers to fathers’ scores *via*, Differences in clinical variables and in the score on the FS-IS-IT questionnaire between fathers and mothers in the presence or absence of feeding/swallowing difficulties were assessed *via* dependent-sample *t-*tests/Wilcoxon’s tests as well.

## Psychometrics

### Face and Content Validity

Face validity (i.e. the degree to which the content of the questionnaire is an adequate reflection of the construct to be measured) and content validity (i.e. the degree to which the items of the questionnaire indeed looks as though they are an adequate reflection of the construct to be measured) [27] of each FS-IS-IT subscale was assessed using mean raters’ scores, with scores ≥ 3.00 considered valid. Such a benchmark was addressed, on an empirical basis, as indexing an acceptable degree of validity (i.e., more than half of the full range of the scale itself, which ranges from 1 to 4).

### Reliability

The FS-IS-IT internal consistency (i.e., the degree of the interrelatedness among the items) and test–retest reliability (i.e., the proportion of the total variance in the measurements that is due to ‘true’ differences between patients) [27] were examined.

Internal consistency was tested, in the group of caregivers of children with feeding/swallowing disorders, *via* McDonald’s ω-statistic, separately for mothers and fathers. Internal consistency was assessed both by addressing all items of the FS-IS-IT and the three subsets of items enclosed within each of its subscale (Subscale 1: items 1–5; Subscale 2: items 6–12; Subscale 3: items 13–18), with values ≥ 0.70 being deemed as acceptable [33]. Test-retest reliability was investigated, in a randomly subsample of *N* = 10 mother-father dyads of children with PFD (*N* = 10 mothers, *N* = 10 fathers) that were followed-up with the FS-IS-IT at a 14-day distance, by means of the intra-class correlation (ICC) coefficient (two-way random effect model) – which was addressed as optimal if ≥ 0.75 [34]. During this period, there was no administration of medication, surgical intervention or behavioral intervention for dysphagia to the children. Furthermore, no counseling intervention from a SLT or psychologist was received by the caregivers. The conditions of the testing, the administering SLT and the environment between the two assessment dates were found to be unchanged. The two-week interval between assessments was deemed sufficient to reduce recall bias and ensure stability between the two assessments.

### Validity

The validity of FS-IS-IT was examined in order to determine the extent to which the FS-IS-IT measures the constructs it claims to measure. For this purpose, the criterion validity (i.e., the degree to which instrument scores adequately reflect a gold standard) and the convergent validity (i.e., the degree of correlation between scores on one scale and those on a comparator scale measuring a related construct) [27] were evaluated. The convergent validity is indeed a form of construct validity, which is the degree to which the scores of an instrument are consistent with hypotheses. To investigate the criterion validity of FS-IS-IT, we chose the Italian version of the ZBI [29] as the gold standard. The rationale underpinning this choice is threefold. Firstly, the instrument boasts satisfactory psychometric properties [35]. Secondly, it has seen extensive utilization in both clinical practice and research studies. Thirdly, and perhaps most crucially, there is a paucity of a dedicated instrument for caregivers of children with disabilities. It is important to note that, although other studies on the validation of FS-IS-IT used the PedsQL™ Family Impact Module (PedsQL™ FIM) [36], a multidimensional instrument used to assess the family’s QoL, this instrument is not translated and validated in Italian. Consequently, previous studies have not investigated the criterion validity of FS-IS-IT based on ZBI, therefore no precise hypotheses on correlational strength could be made. However, according with Prinsen et al. [37], correlations between instruments measuring similar constructs should be ≥ 0.50.

To assess *criterion validity*, a correlation was made between the total score of the FS-IS-IT and the type of diet consumed by the child, as assessed by the IDDSI Functional Diet Scale. Previous studies have not investigated criterion validity; therefore, no precise hypothesis about the strength of the correlation could be formulated. It is important to note that the present sample included children with feeding difficulties who consumed multiple consistencies but had food phobias or very rigid food choices. It is hypothesized that the parents of these children experience elevated levels of stress, and the impact of the disorder is significant, comparable to the parents of children with dysphagia. Consequently, the correlation is hypothesized to be inverse, with a low to moderate strength (i.e. 0.30–0.50, according to Cohen’s [38] interpretation for the magnitude of correlation coefficients: i.e., *r* ≤.30 ◊ small; 0.30 < *r* ≤.50 ◊ medium; *r*_*s*_≥0.50 ◊ large). In line with this hypothesis, and according to Prinsen and colleagues [37], these two instruments measure related but different constructs, and therefore the correlation should be lower.

Validity of both the FS-IS-IT total score and its subscales was tested, in the PFD group, *via* Pearson’s/Spearman’s coefficients against the IDDSI and, separately for mothers and fathers, against the ZBI.

### Diagnostics

*Diagnostic validity* is the most important type of criterion-related validity and refers to the ability of the test to discriminate between individuals with and without a particular disorder (known group validity) [39]. The Area Under the Curve (AUC) was used to assess the diagnostic validity of the FS-IS-IT, i.e. the ability to discriminate between parents of a child with feeding and swallowing problems and parents of a child with developmental problems but without feeding problems and/or dysphagia. According to Swets’ [40] recommendations, AUC values ≥ 0.7 were deemed as acceptable. In addition, different cut-offs are expected for fathers and mothers, based on the idea that mothers should be more stressed.

The diagnostic accuracy of the FS-IS-Total was assessed by means of receiver-operating characteristics (ROC) analyses, separately for mother and fathers, with the positive state being operationalized as the presence of a PFD (PFD+). Sensitivity, specificity, positive and negative predictive values and likelihood ratios were then computed at the optimal cut-off identified by means of Youden’s *J*-statistics.

Analyses were run *via* IBM^®^ SPSS^®^ Statistics 29 (IBM Corp., 2023) and jamovi 2.3 (the jamovi project, 2022). Significance thresholds were adjusted by means of Bonferroni’s approach whenever multiple tests were run (i.e., α_adjusted_ = 0.05/*k*, with *k* being the number of comparisons) in order not to inflate Type-I error rates.

## Results

### Adaptation and Cross-Cultural Validation

The version revised by a group of experts (two speech and language therapists, two psychologists, one cultural-linguistic mediator) resulted in a single pre-tested questionnaire adapted to Italian. In this version, minimal changes were made to favor comprehensibility in Italian. Specifically, in some items, the term “child” was replaced with “son/daughter”, the term “feed” (items 2 and 3) was replaced with “assistance during the meal”, some verbal tenses and modes were modified (items 4, 9, 11), and in item 11 the term “to worry” was replaced with “I wonder with concern”. This version was assessed by a group of deglutological experts, and all items exceeded the pre-specified threshold for both content and face validity scales (Table 1). For this reason, no further modifications were made. Following a thorough analysis of the interview transcripts from the pre-test, it was determined that no difficulties emerged. All participants completed the questionnaire in its entirety and 100% of them expressed no requirement for improvement. Consequently, the final version of the FS-IS-IT in Italian was found to be equivalent to the pre-defined version. The final version of the FS-IS-IT comprises 18 items, which are divided into three subscales (see Appendix). Responses are measured on a Likert scale, ranging from 1 (never) to 5 (almost always). In the event that the item cannot be evaluated, parents are instructed to indicate “not applicable”. The score for each subscale is calculated by adding the Likert scores for the items in that subscale and dividing by the number of items answered in that subscale. The instrument’s total score can be obtained by adding the 18 items and dividing by 18. A higher score indicates a greater impact on quality of life [22]. The total burden score was calculated by adding up the subscale scores (*range* = 0–5), with higher scores indicating a greater level of burden.

**Table 1:** Experts’ content and face validity ratings for FS-IS-IT subscales

	Content validity	Face validity
FS-IS-IT-1	3.4±0.63	3.2±0.58
FS-IS-IT-2	3.4±0.63	3.2±0.78
FS-IS-IT-3	3.1±0.66	3.1±0.73

Notes FS-IS-IT: Feeding/Swallowing-Impact Survey-Italian;. Reported values are *M*±*SD* of experts’ ratings

The average time required to complete the questionnaire was 9.57 min ± 1.17 min.

### Sociodemographic Data and FS-IS-IT

Table 2 summarizes patients’ background and clinical measures. Statistically significant differences were not observed between the group of children with and without PFD with regard to demographic variables (gender, age, birth weight and diagnosis) (see Table 2). The population of children included in the study with PFD was 50% female, with an average age of approximately 4 years. More than 50% of the sample had a genetic diagnosis, slightly less than 30% a neurological disorder and approximately 15% an autistic spectrum disorder. In the group affected by PFD, 75% of the sample was completely dependent at mealtimes, with more than 20% relying on alternative forms of feeding. Conversely, the group of children without PFD did not exhibit a complete dependency on caregivers during mealtimes, with more than 50% demonstrating autonomy. In the group of parents with PFD, it is observed that mothers have statistically higher levels of burden than fathers and are responsible for their children’s mealtimes to a statistically greater extent. Conversely, no such disparities in meal assistence were identified among parents not experiencing PFD (Table 3). A comparison of the two groups reveals that fathers of children with PFD are statistically less involved in managing their children’s mealtimes, but no differences were observed between the mothers of the two groups. The ZBI score of parents with PFD is higher than that of fathers and mothers without symptoms in both subgroups (Table 4).

No association was detected between either mothers’ or fathers’ education and their FS-IS-IT scores (mothers: *r*/*r*_s_≤|0.21|; *p* ≥.193). Overall, no discrepancies were detected between mothers’ and fathers’ ratings on the FS-IS-IT (Table 3).

**Table 2:** Demographic data and characteristics of children with and without PFD and parental dyads measures

	PFD+	PFD-	*p*
N	32	15	-
Age (months)	52.6±34.7 (8-137)	60.1±22.6 (26-104)	0.451^§^
Sex (male/female)	16/16 (50%/50%)	5/10 (33.3%/66.7%)	0.284^*^
Weight at birth (grams)	2526.6±849.6 (370-3670)	2431±1123.2 (500-4625)	0.747^§^
Type of delivery			0.680^*^
Eutocic	15 (%)	8 (%)	
Dystocic	17 (%)	7 (%)	
Diagnosis			0.358^*^
Neurological	9 (28.1%)	3 (20%)	-
Genetic	18 (56.3%)	10 (66.7%)	-
ASD	5 (15.6%)	1 (6.7%)%	-
Congenital	-	1 (6.7%)	-
IDDSI Functional Scale	5.8±1.4 (2-8)	8±0 (8-8)	<0.001^†^
Need for meal assistance			<0.001^*^
Totally independent	2 (6.3%)	8 (53.3%)	-
Partially independent	6 (18.8%)	7 (46.7%)	-
Fully assisted	24 (75%)	-	-
Feed (%)			0.050^*^
NGT or PEG	7 (21.9%)	-	-
Without NGT/PEG	25 (78.1%)	100%	-

Notes ^*^χ^2^-statistics; ^§^*t*-statistics for independent samples; ^†^Mann-Whitney’s *U*-statistics; PFD=pediatric feeding disorder; ASD=autism-*spectrum* disorders; IDDSI= International Dysphagia Diet Standardization Initiative; NGT=nasogastric tube; PEG=percutaneous endoscopic gastrostomy. Categorical variables are reported as frequencies and, in brackets, percentages. Continuous variables are reported as *M*±*SD* and, in brackets, *range*

**Table 3:** Comparison on meal assistance rate, ZBI and FS-IS-IT scores between mothers and fathers in children with (left side) and without PFD (right side)

	PFD+			PFD-		
	Mothers	Fathers	*p*	Mothers	Fathers	*p*
N	32	32		15	15	
Meal assistance (yes/no)	29/3 (90.6%/9.4%)	12/20 (37.5%/62.5%)	<0.001^§^	13/2 (86.7%/13.3%)	11/4 (73.3%/26.7%)	0.361^§^
ZBI	34.9±12.2 (15-70)	29±11.9 (9-52)	0.005^*^	27.1±11.9 (5-45)	16.3±10.1 (1-36)	0.015^†^
FS-IS-IT						
Total	2.6±0.8 (1.4-4.3)	2.4±0.8 (1.2-4.3)	0.290^*^	1.3±0.3 (1-1.9)	1.2±0.3 (1-1.8)	0.722^†^
Subscale 1 - Daily Activity	2.7±1 (1-5)	2.5±1 (1-4.6)	0.221^*^	1.2±0.4 (1-2.2)	1.1±0.2 (1-1.6)	0.581^†^
Subscale 2 - Worries	3.1±0.9 (1.9-5)	2.9±1 (1.1-4.8)	0.201^*^	1.5±0.6 (1-2.7)	1.4±0.6 (1-3)	0.532^†^
Subscale 3 - Feeding Difficulties	1.8±1 (0-5)	1.8±0.8 (1-4.2)	0.540^†^	1±0 (1-1.2)	1.1±0.1 (1-1.5)	0.423^†^

Notes ^*^t-statistics for dependent samples; ^§^χ^2^-statistics for independent samples; ^†^Wilcoxon’s *W*-statistics; ZBI= Zarit Burden Interview; FS-IS-IT=Feeding/Swallowing Impact Survery; PFD=pediatric feeding disorder. Categorical variables are reported as frequencies and, in brackets, percentages. Continuous variables are reported as *M*±*SD* and, in brackets, *range*

**Table 4:** Comparison on meal assistance rate, ZBI and FS-IS-IT scores between mothers of children with vs. without PFD (left side) and fathers of children with vs. without PFD (right side)

	Mothers			Fathers		
	PFD+	PFD-	*p*	PFD+	PFD-	*p*
Meal assistance (%)						
Yes/No	29/3 (90.6%/9.4%)	13/2 (86.7%/13.3%)	0.682*	12/20 (37.5%/62.5%)	11/2 (73.3%/26.7%)	0.022*
ZBI	34.9±12.2 (15-70)	27.1±11.9 (5-45)	0.062^§^	29±11.9 (9-52)	16.3±10.1 (1-36)	0.001^§^
FS-IS-IT						
Total	2.6±0.8 (1.4-4.3)	1.3±0.3 (1-1.9)	<0.001^†^	2.4±0.8 (1.2-4.3)	1.2±0.3 (1-1.8)	<0.001^†^
Subscale 1 - Daily Activity	2.7±1 (1-5)	1.2±0.4 (1-2.2)	<0.001^†^	2.5±1 (1-4.6)	1.1±0.2 (1-1.6)	<0.001^†^
Subscale 2 - Worries	3.1±0.9 (1.9-5)	1.5±0.6 (1-2.7)	<0.001^†^	2.9±1 (1.1-4.8)	1.4±0.6 (1-3)	<0.001^†^
Subscale 3 - Feeding Difficulties	1.8±1 (0-5)	1±0 (1-1.2)	<0.001^†^	1.8±0.8 (1-4.2)	1.1±0.1 (1-1.5)	<0.001^†^

Notes. ^*^χ^2^-statistics; ^§^*t*-statistics for independent samples;; ^†^Mann-Whitney’s *U*-statistics; ZBI= Zarit Burden Interview; FS-IS-IT: Feeding/Swallowing-Impact Survey-Italian; PFD=pediatric feeding disorder. Categorical variables are reported as frequencies and, in brackets, percentages. Continuous variables are reported as *M*±*SD* and, in brackets, *range*

### Psychometrics and Diagnostics

The IDDSI converged with both mothers’ (*r*(32)=-0.41; *p* =.018) and fathers’ (*r*(32)=-0.42; *p* =.018) FS-IS-IT Total scores; similarly, both parents’ ZBI scores was strongly related to their scores on the ZBI (mothers: *r*(32) = 0.73; *p* <.001; fathers: *r*(32) = 0.78; *p* <.001).

The results of the criterion validity and convergent validity analyses for the FS-IS-IT subscales are presented in Table 5: whilst the ZBI moderately-to-strongly converged with both mothers’ and fathers’ scores on each FS-IS-IT subscale, the only significant association that survived Bonferroni’s correction as to the IDDSI was that between this scale and the FS-IS-IT3 in mothers.

The FS-IS-IT showed optimal internal consistency in both mothers (Total: McDonald’s ω = 0.93; item-rest correlations ranging from 0.37 to 0.86; Subscale 1: McDonald’s ω = 0.85; item-rest correlations ranging from 0.55 to 0.74; Subscale 2: McDonald’s ω = 0.86; item-rest correlations ranging from 0.53 to 0.76; Subscale 3: McDonald’s ω = 0.81; item-rest correlations ranging from 0.08 to 0.77) and fathers (Total: McDonald’s ω = 0.94; item-rest correlations ranging from 0.44 to 0.78; Subscale 1: McDonald’s ω = 0.87; item-rest correlations ranging from 0.65 to 0.80; Subscale 2: McDonald’s ω = 0.89; item-rest correlations ranging from 0.258 to 0.75; Subscale 3: McDonald’s ω = 0.86; item-rest correlations ranging from 0.54 to 0.84), also proving to be optimally reliable at re-test (ICC = 0.97).

**Fig. 2:**
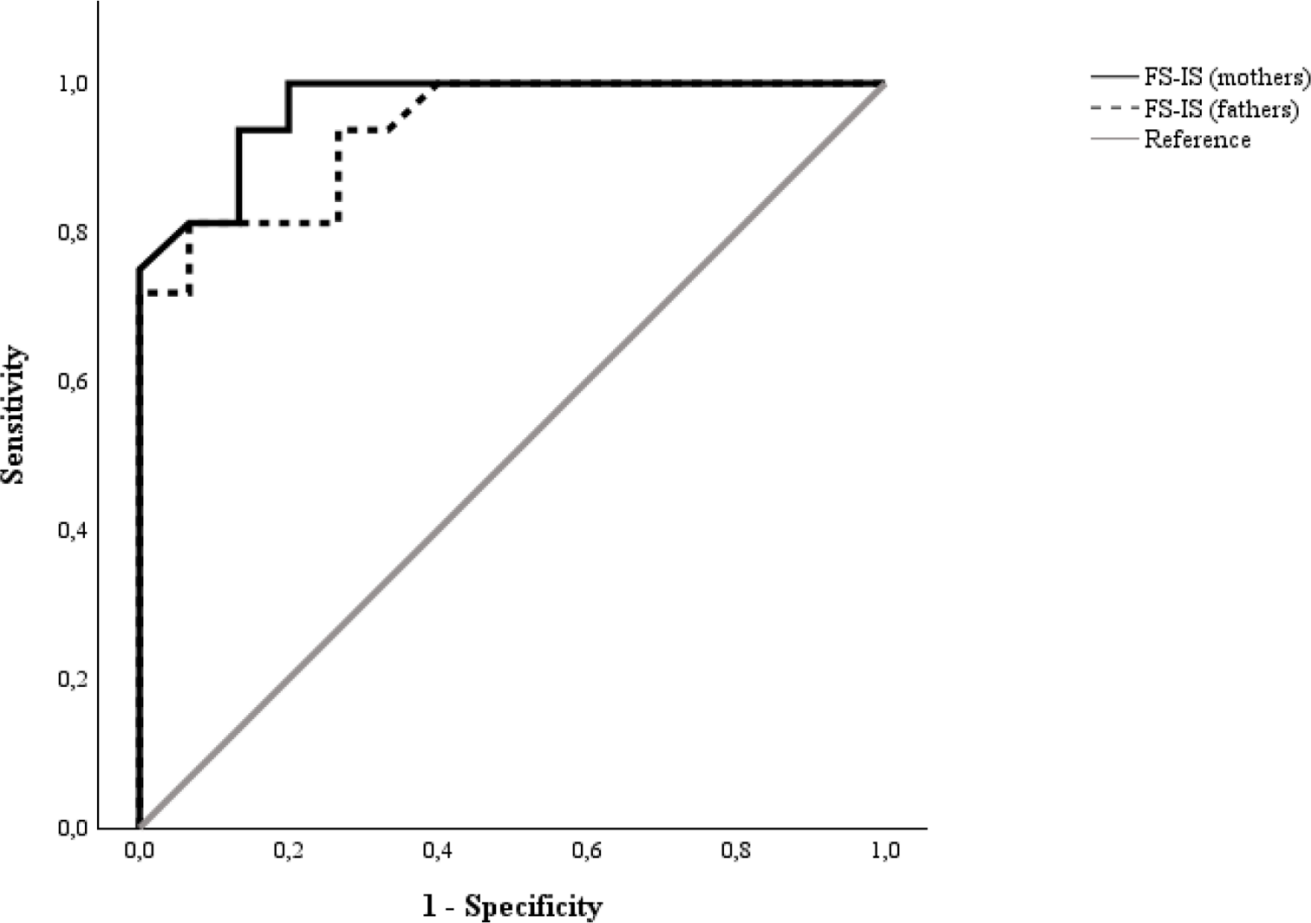
ROC curves for mothers’ and fathers’ FS-IS-IT-Total scores in discriminating PFD + from PFD- children. Notes. ROC = receiver-operating characteristic; FS-IS-IT = Feeding/Swallowing Impact Survey; PFD + = children with pediatric feeding disorders; PFD-=children without pediatric feeding disorders. Mothers: AUC = 0.97; *SE* = 0.02; CI 95% [0.93, 1.00]. Fathers: AUC = 0.94; *SE* = 0.03; CI 95% [0.87, 1.00])

ROC analyses showed that both mothers’ and fathers’ FS-IS-IT-Total scores accurately discriminated PFD + from PFD- children (mothers: AUC = 0.97; *SE* = 0.02; CI 95% [0.93, 1.00]; fathers: AUC = 0.94; *SE* = 0.03; CI 95% [0.87, 1.00]) (Fig. 2). At the optimal cut-offs (mothers: >1.583, *J* = 0.81; fathers: >1.649, *J* = 0.75), the FS-IS proved to be featured by optimal diagnostics (Table 6).

**Table 5:** Construct validity testing results for FS-IS-IT subscales

Mothers			
	FS-IS-IT-1	FS-IS-IT-2	FS-IS-IT-3
IDDSI Functional Scale	*r*=-.39; *p*=.028	*r*=-.30; *p*=.094	*r*_*s*_=-0.47; *p*=.006
ZBI	*r*=.76; *p*<.001	*r*=.65; *p*<.001	*r*_*s*_=0.49; *p*=.004
Fathers			
	FS-IS-IT-1	FS-IS-IT-2	FS-IS-IT-3
IDDSI Functional Scale	*r*=-.40; *p*=.024	*r*=-.28; *p*=.121	*r*_*s*_=-0.39; *p*=.026
ZBI	*r*=.71; *p*<.001	*r*=.69; *p*<.001	*r*_*s*_=0.61; *p*<.001

**Notes** Significant coefficient at α_adjusted_=0.017 are reported in bold; IDDSI= International Dysphagia Diet Standardization Initiative; ZBI= Zarit Burden Interview; FS-IS-IT: Feeding/Swallowing-Impact Survey-Italian

**Table 6:** Diagnostic metrics for mothers’ and fathers’ FS-IS-IT total scores

	Cut-off	J	Se	Sp	PPV	NPV	LR+	LR-
Mothers	1.583	0.81	0.94	0.87	0.93	0.87	7.03	0.07
Fathers	1.649	0.75	0.81	0.93	0.96	0.70	12.19	0.20

Notes FS-IS-IT=Feeding/Swallowing-Impact Survey-Italian; Se=sensitivity; Sp=specificity; PPV=positive predictive value; NPV=negative predictive value; LR+=positive likelihood ratio; LR-=negative likelihood ratio

## Discussion

In the present study, the Feeding/Swallowing Impact Survey (FS-IS) was translated into Italian and cross-adapted according to the guidelines proposed by Beaton et al. [28]. All members of the expert commission involved in the process actively discussed possible solutions to produce a translated instrument that maintained a reading and comprehension level accessible by most respondents, even those with a low level of education, without altering the meaning and content of the original source. For that reason, minimal grammatical or semantic changes were proposed. It is to be noted that the present expert panel did not include anthropologists or sociologists, despite the fact that such inclusion had been recommended by the relevant guidelines [41]. The deglutologist responsible for evaluating the content validity of the scale concluded that the FS-IS-IT was both suitable and equivalent to the original instrument for measuring the impact of PFD in Italian caregivers of children with heterogeneous conditions. Furthermore, during the caregiver testing phase, no problems with item comprehension were reported.

In contrast to previous studies, we included both mother and father for each child. In fact, in line with many studies focusing mainly on the female figure, some validation studies of the FS-IS in other languages have only included mothers [23–24], or fathers are generally underrepresented [22, 25]. However, recent evidence highlights the importance of involving fathers in the care of children with special needs [15]. Therefore, this study aims to verify the impact of PFD on parental dyads, highlighting the importance of involving fathers in the rehabilitation process.

The final version was deemed appropriate and easy to understand, with evidence of reliability and validity for both subgroups (fathers and mothers). Moreover, the study assessed the diagnostic properties of the instrument for the first time. Specific cut-offs were identified to discriminate between parents of children with PFD and parents of children with disabilities but without these symptoms. The results enabled the development of the first valid instrument capable of measuring the impact of PFD on the quality of life of parents of children with disabilities in Italy.

Regarding *reliability analyses*, the FS-IS-IT internal consistency met satisfactory parameters, similar to the previous FS-IS validation studies [22–23, 25]. In contrast to previous studies, which utilized Cronbach’s alpha coefficient, the present study opted to employ McDonald’s Omega coefficient (ω). Indeed, recent evidence suggests that Cronbach’s Alpha coefficient (α) is based on assumptions, whereas McDonald’s Omega coefficient (ω) is based on fewer and more realistic assumptions. Additionally, calculating McDonald’s Omega coefficient (ω) alongside a confidence interval considers variability in the estimation process more closely, resulting in a more precise level of confidence in the consistency of the scale adjustment [42]. Differently from alpha, omega is based on a factor analysis result. As for alpha, internal consistency is usually considered acceptable if the estimate is 0.70 or higher [43]. The ω values obtained for both fathers and mothers could be interpreted as indicating satisfactory internal consistency of the FS-IS-IT. Additionally, the test-retest reliability of the FS-IS-IT was found to be high, indicating its stability and reliability over two weeks. This period reduced recall bias and ensured the stability of patients’ health status between the two assessment sessions.

The *validity* of the FS-IS-IT was established in the present study through the analyses of criterion and convergent validity. Criterion validity was previously evaluated using the PedsQL™ FIM [36], a multidimensional tool for assessing family quality of life. However, this tool has not yet been validated in Italian. Therefore, the Italian version of the ZBI was chosen as the gold standard. This tool was developed based on Zarit’s definition of caregiver burden, which encompasses the extent to which caregivers perceive financial, physical, and spiritual strain. The ZBI is a widely used tool for evaluating caregiver burden and has demonstrated adequate psychometric properties [35]. It has also been utilized in the pediatric field [44–46]. It is important to note that burden should not be equated with quality of life. The latter encompasses positive and negative effects that may coexist for caregivers [47]. However, since the FS-IS-IT questionnaire assesses the impact of a PFD on the caregiver, the ZBI could be considered an appropriate measure.

As expected, moderate to strong statistically significant inverse correlations were observed between the FS-IS-IT and its subscales and the ZBI score in both groups of fathers and mothers. To date, most literature has used the ZBI to assess the burden of dysphagia on caregivers in adulthood and old age, particularly in the context of dementia [48]. In the pediatric setting, it has primarily been used to assess the burden related to enteral nutrition [49]. However, this finding is consistent with previous studies that the presence of PFD is strongly associated with higher levels of burden on caregivers [50–51]. In line with previous studies, caregivers of children with PFD (both fathers and mothers) reported significantly higher levels of burden than caregivers of children without feeding difficulties [52].

Based on the previous hypothesis, the FS-IS-IT total score and subscales have a low-to-mild correlation with the IDDSI Functional Diet Scale. The present analyses showed that only the third subscale in the mothers’ subgroup had a statistically significant correlation with the IDDSI Functional Diet Scale based on Bonferroni correction. This result could be attributed to the lack of sufficient power to find a significant correlation in the present study. Therefore, future studies with larger samples are necessary. Moreover, our sample included children with feeding difficulties who consumed multiple consistencies but had food phobias or very rigid food choices. It is assumed that the parents of these children experience a high level of stress and that the impact of the disorder is significant [53], similar to the parents of children with dysphagia. In accordance with the hypothesis, a recent study by Silverman et al. [54] observed that parents of children who exclusively exhibit problematic mealtime behaviors (e.g., verbal refusal, food avoidance, food selectivity, etc.) despite their children consuming a variety of consistencies reported feelings of parental ineptitude and experienced high levels of stress. However, as the child consumes a variety of consistencies, the IDDSI Functional Diet score is high, thereby limiting the strength of the correlation. Consequently, the present findings suggest that the analysis of the child’s diet alone may not suffice to explain the parental burden experienced. To date, no studies have mapped factors associated with parental burden in case of PFD; however, future studies with a large sample could investigate which factors have the greatest impact. The significant correlation between the IDDSI Functional Diet Scale and the ‘Feeding Difficulties’ subscale in the mothers’ group supports the hypothesis that mothers of children with greater food restrictions experience more practical difficulties in preparing and serving food and drinks. Literature demonstrates that caregivers face challenges during mealtimes, including preparing and offering adequate food and liquids, increased time spent eating, and pressure to provide sufficient food. This evidence corroborates these findings [55, 56]. Furthermore, this result confirms those of previous studies that translated the FS-IS into Brazilian Portuguese [25] and Turkish [23]. These studies demonstrated that the Feeding Difficulties subscale effectively differentiated caregivers of children with and without oral feeding, respectively, or between caregivers of children who aspirated and those who did not. Caregivers face challenges during mealtimes, including preparing and offering adequate food and liquids, increased time spent eating, and pressure to provide sufficient food.

The *diagnostic properties* of the FS-IS-IT has not been previously assessed in validation studies, and no cut-off has yet been investigated. Previous studies have only assessed the discriminant validity between subjects with milder forms of dysphagia and more severe forms, based on instrumental assessments [23] or clinical assessments, such as the presence of parenteral nutrition [22, 25]. Conversely, the present study has showed that the scale can effectively differentiate between parents of children with PFD and parents of children with disabilities but without these difficulties, with high sensitivity and specificity. Additionally, specific cut-off scores for fathers and mothers have been derived for FS-IS-IT. The study found that the FS-IS-IT is a suitable screening tool for identifying PFD in parents of children with disabilities compared to parents of children with disabilities but without PFD. The sensitivity at this point was 94% and 81%, while the specificity was 87% and 93% for the mother and father, respectively. This set of data will prove to be of significant value in clinical practice, and that it constitutes part of the evidence supporting the construct validity of the FS-IS-IT, particularly the known group validity. The study also provides empirical data showing that the quality of life of the caregiver of a child with PFD is impaired. However, it should be noted that the distribution of medical diagnoses and impairments in the study population is reflective of the population of the Centre where the study was conducted. Consequently, these distributions cannot be extrapolated to the PFD population. Therefore, a larger study with different populations and ages should be conducted to confirm our results. Furthermore, the burden should be influenced by culture, especially for topics related to eating and meals. Therefore, studies conducted in different countries should be useful in investigating the different impact of culture.

As hypothesized, fathers were found to be less involved in their children’s feeding than mothers in both groups of parents of children with disabilities, with or without PFD, although no statistical difference was observed between dyads in the same groups at FS-IS-IT scores. Surprisingly, the percentage of fathers involved in feeding management is statistically significantly greater in the non-PFD group than in the other group (X-squared = 4.8763, df = 1, p-value = 0.02723). It is noteworthy that fathers of children with disabilities but without PFD were involved in their children’s feeding in about 80% of cases. This finding is consistent with recent research showing that fathers are increasingly involved in their children’s care [57]. Additionally, the definition of fatherhood has expanded to encompass traditionally considered feminine activities [58].

In contrast, when it comes to the group of children with PFD, only about 40% of fathers report taking care of their children’s feeding, leaving the mother as the primary caregiver. To understand the reasons for this disparity, it would be appropriate to conduct studies with larger samples using qualitative methods such as interviews and focus groups to explore the underlying motivations in depth. Mothers may be more involved in the care of their children with PFD due to their greater knowledge of feeding procedures. Although fathers are increasingly involved in housework and childcare, they are still not significantly involved in their children’s rehabilitation. Therefore, fathers may have less information and strategies to deal with feeding difficulties, leaving the task to the mother figure.

However, contrary to the initial hypothesis, the study found no statistically significant difference between the total score of the FS-IS-IT and its subscales obtained from fathers and mothers of children with PFD. This suggests that PFD have a comparable impact on both members of the dyad. Parents face similar challenges in their daily lives and share concerns about their children’s health. They also experience comparable difficulties in managing mealtimes and feeding. Child health professionals and services should sensitize and prepare health teams, including all stakeholders, to welcome and value male participation. This includes involving fathers in circles of discussion, reflection, and sharing of experiences and concerns. This could lead to a more equitable sharing of childcare responsibilities, with fathers feeling more engaged and willing, and could reduce the burden on both parents. Indeed, a growing body of literature has demonstrated the need for holistic family-centered approaches that ensure the care of the whole family in order to reduce the burden of care. Parents with adequate resources and support, as well as physical and psychological health, are able to provide a positive caregiving environment, which is therefore fundamental to the success of habilitative interventions for the child. Involving both fathers and mothers may also facilitate the generalization of skills learned in the therapeutic setting to other life contexts [59].

Finally, the present study found that the impact of a child’s PFD on the caregiver was greater than that reported in the original article [22], but as observed by Simione et al. [60], total scores ranged from 1.9 to 3.3, which is similar to other FS-IS validation studies [25]. As suggested by Rama et al. [25] and Simione and colleagues [60], these discrepancies may be due to differences in the children’s age, varying study populations, and the survey’s timing. In contrast to the original study, we enrolled older children with different aetiologies, including autism. The median age of the children in our study was 52 months, compared to 14 months in the original study. We also included patients at the time of their initial assessment and during follow-up. Moreover, contrary to our expectations and a previous study [25], we did not find any significant relationship between FS-IS-IT scores and education levels. Therefore, it can be hypothesized that the observed variability in total scores on the FS-IS may be ascribed to a number of factors, including the etiology of the feeding impairment, the age group, and the demographic characteristics of the caregivers, amongst others. Regrettably, we were also unable to undertake comparative analyses of differences between the groups with different medical diagnoses, age ranges, or impairments due to insufficient statistical power. Consequently, the implementation of a future study involving a large sample of caregivers of children with PFD who exhibit diverse characteristics (e.g., age, diagnosis, diet, etc.) may contribute to the enhancement of the tool’s construct validity.

### Limitations

The present study is not without limitations. The relatively small sample size calls for caution when interpreting the results, especially since it is the first study to analyze the psychometric properties of the FS-IS for both mothers and fathers. Additionally, the lack of instrumental assessment of swallowing as a construct validity is a limitation of this study. Regrettably, numerous patients lack access to these assessment methods and must rely on clinical diagnosis. Furthermore, only a small proportion of children experienced significant limitations in their diet consistency or alternative feeding channels. In addition, children with congenital craniofacial malformations, which can be associated with feeding and swallowing difficulties, are under-represented in our sample.

Finally, the lack of factor and response analyses should be acknowledged as a limitation of the present study, as no claims can be made about the construct(s) underlying FS-IS-IT and its ability to reflect clinically significant changes over time on the basis of the present results. It is important to note that a sample size of at least 100 participants with 4–10 participants per item is required to conduct factor analyses [61]. Although our study included over 90 carers, as with other validation studies [23, 25], the presence of clusters (dyads) requires a large number of subjects. Future research studies with larger samples of carers of children with mild to relevant feeding and swallowing problems from different aetiologies are needed to confirm the results of the present study and enhance new results on the psychometric properties of the FS-IS-IT scale.

## Conclusion

FS-IS-IT is a reliable, easy-to-use and valid tool for examining the impact of a child’s feeding and swallowing difficulties on both fathers and mothers. The FS-IS-IT demonstrated strong overall reliability and criterion validity. Furthermore, it is a valuable tool for identifying parents of children with PFD with a high level of sensitivity and specificity, distinguishing them from parents of children with disabilities but without these issues. The study identified a cut-off for fathers’ and mothers’ total scores. Furthermore, the study found no difference in the impact of PFD between the two members of the parental dyads. This evidence highlights the importance of involving both parents in assessing, treating, and counselling their children’s PFD. Future studies should conduct factor and responsiveness analyses.

## Appendix

Final version of the IT-FS-IS. Italian-translated items are reported in italics.

Feeding/Swallowing Impact Survey (FS-IS).

Italian Feeding/Swallowing Impact Survey (FS-IS-IT).

## References

[1] Goday PS, Huh SY, Silverman A, Lukens CT, Dodrill P, Cohen SS, Delaney AL, Feuling MB, Noel RJ, Gisel E, Kenzer A, Kessler DB, de Kraus O, Browne J, Phalen JA. Pediatric feeding disorder: consensus definition and conceptual framework. J Pediatr Gastroenterol Nutr. 2019;68(1):124–9. 10.1097/MPG.0000000000002188. 30358739 10.1097/MPG.0000000000002188 PMC6314510

[2] Lefton-Greif MA, Arvedson JC. Pediatric feeding/swallowing: yesterday, today, and tomorrow. Semin Speech Lang. 2016;37(4):298–309. 10.1055/s-0036-1587702. 27701706 10.1055/s-0036-1587702

[3] Arvedson J, Clark H, Lazarus C, Schooling T, Frymark T. The effects of oral-motor exercises on swallowing in children: an evidence-based systematic review. Dev Med Child Neurol. 2010;52(11):1000–13. 10.1111/j.1469-8749.2010.03707.x. 20497451 10.1111/j.1469-8749.2010.03707.x

[4] Linscheid TR, Budd KS, Rasnake LK. Pediatric feeding disorders. In: Roberts MC, editor. Handbook of pediatric psychology. New York: Guilford Press; 2003. pp. 481–98.

[5] Ancel PY, Livinec F, Larroque B, et al. Cerebral palsy among very preterm children in relation to gestational age and neonatal ultrasound abnormalities: the EPIPAGE cohort study. Pediatrics. 2006;117:828–35. 10.1542/peds.2005-0091. 16510664 10.1542/peds.2005-0091

[6] Bhattacharyya N. The prevalence of pediatric voice and swallowing problems in the united States. Laryngoscope. 2015;125(3):746–50. 10.1002/lary.24931. 25220824 10.1002/lary.24931

[7] Hamilton KE, Redshaw ME, Tarnow-Mordi W. Nurse staffing in relation to risk-adjusted mortality in neonatal care. Arch Dis Child Fetal Neonatal Ed. 2007;92(2):F99–103. 10.1136/adc.2006.102988. 17088341 10.1136/adc.2006.102988 PMC2675478

[8] Jadcherla S. Dysphagia in the high-risk infant: potential factors and mechanisms. Am J Clin Nutr. 2016;103(2):S622–8. 10.3945/ajcn.115.110106. 10.3945/ajcn.115.110106 PMC4733255 26791178

[9] Arvedson JC, Brodsky L, Lefton-Greif MA. Pediatric swallowing and feeding assessment and management. CA: Plural Publishing, San Diego; 2020.

[10] Williams KE, Seiverling L. Assessment and treatment of feeding problems among children with autism spectrum disorders. Comprehensive guide to autism. New York: Springer; 2014. pp. 1973–93.

[11] Rodenburg R, Meijer AM, Dekovic M, Aldenkamp AP. Parents of children with enduring epilepsy: predictors of parenting stress and parenting. Epilepsy Behav. 2007;11:197–207. 17604228 10.1016/j.yebeh.2007.05.001

[12] Wallander JL, Varni JW. Effects of pediatric chronic physical disorders on child and family adjustment. J Child Psychol Psychiatry Allied Discip. 1998;39(1):29–46. 9534085

[13] Raina P, O’Donnell M, Rosenbaum P, Brehaut J, Walter SD, Russell D, Swinton M, Zhu B, Wood E. The health and well-being of caregivers of children with cerebral palsy. Pediatrics. 2005;115(6):e626–36. 10.1542/peds.2004-1689. 15930188 10.1542/peds.2004-1689

[14] Liley AJ, Manthorpe J. The impact of home enteral tube feeding in everyday life: a qualitative study. Health Soc Care Community. 2003;11(5):415–22. 10.1046/j.1365-2524.2003.00444.x. 14498838 10.1046/j.1365-2524.2003.00444.x

[15] Uribe-Morales BM, Cantero-Garlito PA, Cipriano-Crespo C. Fathers in the care of children with disabilities: an exploratory qualitative study. Healthc (Basel). 2021;10(1):14. 10.3390/healthcare10010014. 10.3390/healthcare10010014 PMC8775232 35052178

[16] Crowe TK, VanLeit B, Berghmans KK. Mothers’ perceptions of child care assistance: the impact of a child’s disability. Am J Occup Ther. 2000;54:52–8. 10.5014/ajot.54.1.52. 10686627 10.5014/ajot.54.1.52

[17] Cantero-Garlito PA, Moruno-Miralles P, Flores-Martos JA. Mothers who take care of children with disabilities in rural areas of a Spanish region. Int J Environ Res Public Health. 2020;17(8):2920. 10.3390/ijerph17082920. 32340226 10.3390/ijerph17082920 PMC7215576

[18] Pleck JH, Masciadrelli BP. Paternal involvement by U.S. Residential fathers: levels, sources, and consequences. In: Lamb ME, editor. The role of the father in child development. 4th ed. John Wiley & Sons, Inc.; 2004. pp. 222–71.

[19] Bourke-Taylor H, Howie L, Law M. Impact of caring for a school-aged child with a disability: Understanding mothers’ perspectives. Aust Occup Ther J. 2010;57(2):127–36. 10.1111/j.1440-1630.2009.00817.x. 20854578 10.1111/j.1440-1630.2009.00817.x

[20] Ranehov L, Håkansson C. Mothers’ experiences of their work as healthcare assistants for their chronic disabled child. Scand J Occup Ther. 2019;26(2):121–34. 10.1080/11038128.2018.1483427. 29983089 10.1080/11038128.2018.1483427

[21] Duran S, Duran S, Acunas B, Cesur G, Ciftdemir NA. Eating behaviors of late and moderately preterm infants at two years of age and their associations with mothers’ mental health. J Pediatr Gastroenterol Nutr. 2020. 10.1097/MPG.0000000000002947. 10.1097/MPG.0000000000002947 32960828

[22] Lefton-Greif MA, Okelo SO, Wright JM, Collaco JM, McGrath-Morrow SA, Eakin MN. Impact of children’s feeding/swallowing problems: validation of a new caregiver instrument. Dysphagia. 2014;29(6):671–7. 10.1007/s00455-014-9560-7. 25159316 10.1007/s00455-014-9560-7 PMC4359894

[23] Arslan S, Kılınç H, Yaşaroğlu Ö, İnal Ö, Demir N, Karaduman A. Reliability and validity of the Turkish version of the feeding/ swallowing impact survey. J Dev Phys Disabil. 2018. 10.1007/s10882-018-9615-z.

[24] Mokhlesin M, Ebadi A, Yadegari F, Ghoreishi ZS. Translation and psychometric properties of the Persian version of the feeding/swallowing impact survey in Iranian mothers. Folia Phoniatr Logop. 2024;76(1):22–9. 10.1159/000531023. 37231856 10.1159/000531023

[25] Rama CG, Bernardes FB, Lefton-Greif MA, Levy DS, Bosa VL. Translation, cultural adaptation, reliability, and validity evidence of the feeding/swallowing impact survey (FS-IS) to Brazilian Portuguese. Dysphagia. 2022;37(5):1226–37. 10.1007/s00455-021-10383-4. 34779911 10.1007/s00455-021-10383-4

[26] Fracchia MS, Diercks G, Yamasaki A, Hersh C, Hardy S, Hartnick M, Hartnick C. Assessment of the feeding swallowing impact survey as a quality-of-life measure in children with laryngeal cleft before and after repair. Int J Pediatr Otorhinolaryngol. 2017;99:73–7. 28688569 10.1016/j.ijporl.2017.05.016

[27] Mokkink LB, Terwee CB, Patrick DL, et al. The COSMIN study reached international consensus on taxonomy, terminology, and definitions of measurement properties for health-related patient-reported outcomes. J Clin Epidemiol. 2010;63(7):737–45. 10.1016/j.jclinepi.2010.02.006. 20494804 10.1016/j.jclinepi.2010.02.006

[28] Chattat R, Cortesi V, Izzicupo F, et al. The Italian version of the Zarit burden interview: a validation study. Int Psychogeriatr. 2011;23(5):797–805. 10.1017/S1041610210002218. 21205379 10.1017/S1041610210002218

[29] Steele CM, Namasivayam-MacDonald AM, Guida BT, et al. Creation and initial validation of the international dysphagia diet standardisation initiative functional diet scale. Arch Phys Med Rehabil. 2018;99(5):934–44. 10.1016/j.apmr.2018.01.012. 29428348 10.1016/j.apmr.2018.01.012 PMC5961739

[30] Cichero JAY, Lam PTL, Chen J, et al. Release of updated international dysphagia diet standardisation initiative framework (IDDSI 2.0). J Texture Stud. 2020;51(1):195–6. 10.1111/jtxs.12481. 31498896 10.1111/jtxs.12481

[31] Beaton DE, Bombardier C, Guillemin F, Bosi Ferraz M. Guidelines for the process of cross-cultural adaptation of self-report measures. Spine (Phila Pa 1976). 2000;25:3186–91. 11124735 10.1097/00007632-200012150-00014

[32] Kim HY. Statistical notes for clinical researchers: assessing normal distribution (2) using skewness and kurtosis. Restor Dentistry Endodontics. 2013;38(1):52–4. 10.5395/rde.2013.38.1.52. 10.5395/rde.2013.38.1.52 PMC3591587 23495371

[33] McNeish D. Thanks coefficient alpha, we’ll take it from here. Psychol Methods. 2018;23(3):412–33. 10.1037/met0000144. 28557467 10.1037/met0000144

[34] Koo TK, Li MY. A guideline of selecting and reporting intraclass correlation coefficients for reliability research. J Chiropr Med. 2016;15(2):155–63. 10.1016/j.jcm.2016.02.012. 27330520 10.1016/j.jcm.2016.02.012 PMC4913118

[35] Yu Y, Liu ZW, Li TX, Zhou W, Xi SJ, Xiao SY, Tebes JK. A comparison of psychometric properties of two common measures of caregiving burden: the family burden interview schedule (FBIS-24) and the Zarit caregiver burden interview (ZBI-22). Health Qual Life Outcomes. 2020;18:1–9. 10.1186/s12955-020-01335-x. 32252766 10.1186/s12955-020-01335-x PMC7137330

[36] Varni JW, Sherman SA, Burwinkle TM, Dickinson PE, Dixon P. The PedsQL family impact module: preliminary reliability and validity. Health Qual Life Outcomes. 2004;2:55. 10.1186/1477-7525-2-55. 15450120 10.1186/1477-7525-2-55 PMC521692

[37] Prinsen CAC, Mokkink LB, Bouter LM, Alonso J, Patrick DL, de Vet HCW, Terwee CB. Qual Life Res. 2018;27(5):1147–57. COSMIN guideline for systematic reviews of patient-reported outcome measures. 10.1007/s11136-018-1798-3 10.1007/s11136-018-1798-3 PMC5891568 29435801

[38] Cohen J. A power primer. Psychol Bull. 1992;112(1):155–57. 19565683 10.1037//0033-2909.112.1.155

[39] Rodrigues IB, Adachi JD, Beattie KA, Lau A, MacDermid JC. Determining known-group validity and test-retest reliability in the PEQ (personalized exercise questionnaire). BMC Musculoskelet Disord. 2019;20:1–10. 31412834 10.1186/s12891-019-2761-3 PMC6694546

[40] Swets JA. Measuring the accuracy of diagnostic systems. Volume 240. Science (New York; 1988. pp. 1285–93. 485710.1126/science.3287615. 10.1126/science.3287615 3287615

[41] Terwee CB, Prinsen C, Chiarotto A, De Vet H, Bouter LM, Alonso J, Mokkink LB. COSMIN methodology for assessing the content validity of PROMs–user manual. Amsterdam: VU University Medical Center; 2018. pp. 1159–70. 10.1007/s11136-018-1829-0 PMC5891557 29550964

[42] Bonniga R, Saraswathi DA. (2020). Literature review of cronbachalphacoefficient and mcdonald’s omega coefficient. Eur J Mol Clin Med, 7(06).

[43] Cicchetti DV. Guidelines, criteria, and rules of thumb for evaluating normed and standardized assessment instruments in psychology. Psychol Assess. 1994;6(4):284.

[44] Boluarte-Carbajal A, Paredes-Angeles R, Tafur-Mendoza AA. Psychometric properties of the Zarit burden interview in informal caregivers of persons with intellectual disabilities. Front Psychol. 2022;13:792805. 10.3389/fpsyg.2022.792805. 35356334 10.3389/fpsyg.2022.792805 PMC8959923

[45] Purpura G, Tagliabue L, Petri S, Cerroni F, Mazzarini A, Nacinovich R. Caregivers’ burden of School-Aged children with neurodevelopmental disorders: implications for Family-Centred care. Brain Sci. 2021;11(7):875. 10.3390/brainsci11070875. 34208983 10.3390/brainsci11070875 PMC8301981

[46] Gbolahan OO, Amiede OS, Samuel OA. The burden and perceived stress on family caregivers of patients with orofacial cleft deformities in the perioperative period of cleft repair. J Patient Exp. 2020;7(6):1602–9. 10.1177/2374373520948650. 33457620 10.1177/2374373520948650 PMC7786686

[47] Martin MP, McEntee ML, Suri Y. Caregiver quality of life: how to measure it and why. Am J Health Promot. 2021;35(7):1042–5. 10.1177/08901171211030142f. 34351244 10.1177/08901171211030142f

[48] Rangira D, Najeeb H, Shune SE, Namasivayam-MacDonald A. Understanding burden in caregivers of adults with dysphagia: A systematic review. Am J speech-language Pathol. 2022;31(1):486–501. 10.1044/2021_AJSLP-21-00249. 10.1044/2021_AJSLP-21-00249 34962832

[49] Suluhan D, Yildiz D, Surer I, Fidanci Eren B, Balamtekin N, Nutrition. 36(6), 1220–9. 10.1002/ncp.10586 10.1002/ncp.10586 33047836

[50] Simione M, Dartley AN, Cooper-Vince C, Martin V, Hartnick C, Taveras EM, Fiechtner L. Family-centered outcomes that matter most to parents: A pediatric feeding disorders qualitative study. J Pediatr Gastroenterol Nutr. 2020;71(2):270–5. 10.1097/MPG.0000000000002741. 32304556 10.1097/MPG.0000000000002741 PMC8204401

[51] Hewetson R, Singh S. The lived experience of mothers of children with chronic feeding and/or swallowing difficulties. Dysphagia. 2009;24(3):322–32. 10.1007/s00455-009-9210-7. 19259730 10.1007/s00455-009-9210-7

[52] Dodrill PHH. In: McMurray SMRJ, editor. Quality of life assessment in children with feeding and swallowing disorders. Multidisciplinary Management of Pediatric Voice and Swallowing Disorders. Springer; 2020.

[53] Tereshko L, Weiss MJ, Olive ML. Ethical considerations of behavioral feeding interventions. Behav Anal Pract. 2021;14(4):1157–68. 10.1007/s40617-021-00559-7. 34868819 10.1007/s40617-021-00559-7 PMC8586383

[54] Silverman AH, Erato G, Goday P. The relationship between chronic paediatric feeding disorders and caregiver stress. J Child Health Care. 2021;25(1):69–80. 10.1177/1367493520905381. 32048866 10.1177/1367493520905381

[55] Estrem HH, Thoyre SM, Knafl KA, Frisk Pados B, Van Riper M. It’s a long-term process: description of daily family life when a child has a feeding disorder. J Pediatr Health Care. 2017;32(4):340–7. 10.1016/j.pedhc.2017.12.002. 10.1016/j.pedhc.2017.12.002 PMC6026064 29395666

[56] Polack S, Adams M, O’banion D, et al. Children with cerebral palsy in Ghana: malnutrition, feeding challenges, and caregiver quality of life. Dev Med Child Neurol. 2018;60(9):914–21. 10.1111/dmcn.13797. 29736993 10.1111/dmcn.13797

[57] Raley S, Bianchi SM, Wang W. (2012) When Do Fathers Care? Mothers’ Economic Contribution and Fathers’ Involvement in Child Care. 117(5):1422–1459. 10.1086/663354 10.1086/663354 PMC4568757 26379287

[58] Lamb ME. The role of the father in the child development. New York: Wiley; 2010.

[59] King S, Teplicky R, King G, Rosenbaum P. Family-centered service for children with cerebral palsy and their families: a review of the literature. Semin Pediatr Neurol. 2004;11(1):78–86. 10.1016/j.spen.2004.01.009. 15132256 10.1016/j.spen.2004.01.009

[60] Simione M, Harshman S, Cooper-Vince CE, Daigle K, Sorbo J, Kuhlthau K, Fiechtner L. Examining health conditions, impairments, and quality of life for pediatric feeding disorders. Dysphagia. 2023;38(1):220–6. 10.1007/s00455-022-10455-z. 35486189 10.1007/s00455-022-10455-z PMC9616965

[61] Terwee CB, Bot SDM, de Boer MR, Knol DL, Dekker J, Bouter LM, de Vet HC. Quality criteria were proposed for measurement properties of health status questionnaires. J Clin Epidemiol. 2007;60(1):34–42. 10.1016/j.jclinepi.2006.03.012. 17161752 10.1016/j.jclinepi.2006.03.012

